# *Salmonella* Promotes Its Own Survival in B Cells by Inhibiting Autophagy

**DOI:** 10.3390/cells11132061

**Published:** 2022-06-29

**Authors:** Lopez-Bailon Luis, Gonzalez-Telona Ana, Galán-Enríquez Carlos, García-Gil Abraham, Estrada-García Iris, Moreno-Lafont Martha, Ortiz-Navarrete Vianney

**Affiliations:** 1 Departamento y Posgrado en Inmunología, Escuela Nacional de Ciencias Biológicas del Instituto Politécnico Nacional (ENCB-IPN), Mexico 11350, Mexico; llopezb1002@alumno.ipn.mx (L.-B.L.); iestrada5@hotmail.com (E.-G.I.); mlafont@ipn.mx (M.-L.M.); 2 Departamento de Biomedicina Molecular, Centro de Investigación y de Estudios Avanzados del Instituto Politécnico Nacional, Mexico 07360, Mexico; ana.gonzalezt@cinvestav.mx (G.-T.A.); cgalane@outlook.com (G.-E.C.); garciagilabraham@gmail.com (G.-G.A.)

**Keywords:** *Salmonella*, B cells, autophagy, SopB, mTORC1, ULK1

## Abstract

*Salmonella* is a Gram-negative bacterium known to be the major cause of gastrointestinal diseases and systemic infections. During infection of murine B cells, *Salmonella* activates the PI3K/Akt pathway through its effector, SopB. This signaling pathway induces the downregulation of NLRC4 transcription, resulting in reduced secretion of IL-1β. Thus, *Salmonella*-infected B cells do not progress to pyroptosis; consequently, the bacteria can survive inside these cells. However, the mechanism by which *Salmonella* evades the control of B cells has not yet been elucidated. In this study, we found that SopB activates mTORC1, which is necessary for bacterial survival, since B cells cultured with the mTORC1 inhibitor rapamycin and B cells lacking raptor can control *Salmonella* infection. A similar result was observed in B cells when they were infected with the *Salmonella* SopB mutant (Δsopb). *Salmonella* also promoted the phosphorylation of the ULK1 complex at serine 757 (Ser757) by mTORC1, resulting in decreased levels of LC3-II in infected B cells. In this study, we did not observe these results when B cells were infected with Δsopb *Salmonella*. Our results demonstrated that *Salmonella* survival within B cells depends on the inhibition of autophagy by mTORC1 activation.

## 1. Introduction

*Salmonella enterica* serovar Typhimurium (*Salmonella*) is a Gram-negative bacterium that causes gastrointestinal illness in humans, whereas in mice it causes a systemic infection similar to typhoid fever [1]. Worldwide, it is estimated that between 11 and 20 million new infections occur annually [2]. In addition, approximately 2–5% of people who become ill with typhoid fever become carriers of *Salmonella* [3]. In the gallbladder of these individuals, *Salmonella* forms biofilms that have been associated with the formation of gallstones and bladder stones, allowing *Salmonella* to evade both the immune system and the effect of drugs [4,5]. Once ingested, the bacteria cross the epithelial barrier of the small intestine, where they infect macrophages. These infected cells die by pyroptosis as a consequence of inflammasome activation, which permits *Salmonella* to spread to other anatomical sites and infect other cells [1,6]. Among the cells that may be infected are B cells, which have been reported to allow *Salmonella* survival for at least 60 days [7,8,9].

During the process of B-cell infection, *Salmonella* translocates a series of effector proteins through its Type III secretion system (T3SS). Among these effector proteins is SopB. Together with SopE and SopE2, SopB is involved in the activation of Cdc42 and Rac, permitting the entry of *Salmonella* into nonphagocytic cells [10,11]. In addition, SopB induces the accumulation of PIP3 in the cell membrane via its inositide phosphatase function, leading to the activation of PI3K [12]. During B-cell infection, SopB promotes Akt activation mediated by PI3K and thus the activation of the PI3K/Akt pathway, which allows phosphorylation and retention of YAP in the cytoplasm of B cells, preventing its dimerization with p73 in the nucleus; hence, NLRC4 gene expression does not occur [13,14]. Consequently, *Salmonella* represses inflammasome activation and IL-1β production. Contrary to what is observed in macrophages, B cells are not killed by pyroptosis; thus, *Salmonella* is able to survive within these cells. Pharmacological inhibitors for PI3K (wortmannin), AKT (AKTVII), or PDK1 (GSK 23334470) restore IL-1β production and bacterial control in B cells. Likewise, B lymphocytes from rictor gene conditional knockout mice also produce IL-1β and efficiently control *Salmonella* infection [14]. However, the mechanism that prevents this cell lineage from controlling *Salmonella* infection has not been described.

One of the main mechanisms for eliminating intracellular pathogens is autophagy [15,16]. This mechanism is characterized by the formation of double-membrane vacuoles, called autophagosomes. The onset of autophagosome formation is dependent on the ULK1 complex, which consists of UKL1, FIP200, and ATG101 proteins [17]. During autophagosome maturation, LC3-I binds to the autophagosome membrane to form LC3-II, allowing the recruitment of cargo into the phagosomes, which subsequently fuse with lysosomes, where enzymes degrade the content [18]. mTORC1 and AMPK are central regulators of the ULK1 complex. AMPK allows the activation of ULK1, whereas mTORC1 activation inhibits ULK1 by phosphorylating its serine 757 (Ser757) [17,19,20]. Although autophagy is a mechanism that effectively eliminates intracellular pathogens, several bacteria have adapted various mechanisms that allow them to evade it [16,21,22,23].

Since the PI3K/Akt pathway activates mTORC1 and this pathway is active during B-cell *Salmonella* infection, we considered whether *Salmonella* survival within B cells is autophagy inhibition-dependent through the activation of the PI3K/Akt/mTORC1 pathway through SopB.

## 2. Materials and Methods

### 2.1. Salmonella Strains

*Salmonella enterica* serovar Typhimurium 14028 (*Salmonella*) was obtained from ATCC, which was used to generate a strain lacking SopB (*Salmonella* Δ*sopb*), using the lamda red system, as previously described. *Salmonella* and *Salmonella* Δ*sopb* were transformed with the plasmid pEM180 [24], which codes for a green fluorescent protein (GFP).

*Salmonella* Δ*sopb* was reconstituted with pSopBWT, a plasmid that expresses SopB under its promoter [25], or pSopBC460S, a plasmid that codifies for an inactive catalytic form of SopB [26]. Both the GFP and plasmid-reconstituted *Salmonella* strains were grown in LB medium supplemented with ampicillin (100 μg/mL).

### 2.2. Mice

C57BL/6 mice were obtained from the CINVESTAV Experimental Animal Production Unit; cd19^+/cre^raptor^fl/fl^ mice were generated by performing several crosses between B6.Cg-Rptor^tm1.1Dmsa^/J (Jackson Laboratory 013188) and B6.129P2(C)-Cd19^tm1(cre)Cgn^/J (Jackson Laboratory 006785) mice. The genotype of these animals was corroborated according to the Jackson Laboratory’s instructions. Mice were handled according to NOM-062-ZOO-1999 and CINVESTAV guidelines. This project was approved by CINVESTAV’s Animal Care and Use Committee under protocol 0078-14.

### 2.3. Primary B-Cell Culture

Primary B cells from 6-to-8-week-old C57BL/6 mouse spleens were purified through negative selection, according to the manufacturer’s protocol (Miltenyi Biotec 130.090.862). The percentage of purity was evaluated through flow cytometry. A purity of about 98% was obtained (Figure S1A). Primary B cells were grown in RPMI medium supplemented with 10% heat-inactivated fetal bovine serum (RPMI 10% FBS) at 37 °C in 5% CO_2_.

### 2.4. Infection of B Cells with Salmonella

*Salmonella* strains were grown in LB medium supplemented with their corresponding antibiotics at 37 °C, with agitation for 18 h. Then, a dilution of 1:30 was performed in fresh LB medium, which was then incubated at 37 °C until a density of 0.6 was obtained at 600 nm. Subsequently, bacteria were centrifuged and then resuspended in RPMI 10% FBS. B cells were placed in contact with *Salmonella* at a MOI of 50 for 10 min at 37 °C; after incubation, the cells were centrifuged and the supernatant was discarded. The pellet alone was incubated for 20 min at 37 °C in 5% CO_2_. After this, cells were washed twice with PBS-gentamycin (80 μg/mL) and cultivated in RPMI 10% FBS supplemented with gentamycin (80 μg/mL) at 37 °C in 5% CO_2_. The percentage of infected B cells oscillated between 12% and 20% (Figure S1B). At 1, 3, and 24 h post-infection, cells were harvested through centrifugation, and the formed pellet was lysed using PBS 1% Triton X-100. Dilutions of lysates were plated in LB agar to measure CFUs. For pharmacological inhibitors essays, these were added 1 h before the infection and were maintained during the infection process. Pharmacological inhibitors were used at the following concentrations: wortmannin (Sigma, St. Louis, MO, USA, W1628), 0.05, 0.2, and 1.0 μM; AZD8186 (Selleck, S7694), 500 nM; and rapamycin (Sigma, St. Louis, MO, USA, R0395), 160 nM.

*Salmonella*-infected primary B cells were cultured for 24 h in RPMI medium supplemented with 10% FBS. Peptide D11 (Novus, NBP2-49888) and a control scramble peptide, L11 (Novus, NBP2-49887), were added after 21.5 h post-infection at a concentration of 20 μM, followed by the growth of the cultured cells at 24 h post-infection. Formation of CFUs was evaluated 24 h post-infection through plating on LB agar.

### 2.5. Viability Assays on Salmonella and B Cells

Cytotoxicity was assessed using a lactate dehydrogenase (LDH) release assay (Promega, G1780). The percentage of LDH released was calculated using the following formula: percentage of release = (experimental LDH release—spontaneous LDH release)/(maximal LDH release—spontaneous LDH release) × 100%. To evaluate the effect of the inhibitors in *Salmonella*, a kinetic assay was conducted. Inhibitor concentrations were maintained, as previously shown. Assays were performed over the 8 h; for each hour, the optic density was evaluated. At the end of the lapse time, CFU formation was evaluated through plating on LB agar.

### 2.6. Western Blot Analysis

Total infected B-cell protein was obtained through lysis with RIPA buffer. Samples in acrylamide gel were resolved at 12% or 15% via SDS-PAGE. Subsequently, proteins were transferred to a PVDF membrane and then blocked using 1% BSA solution for 1 h. Antibodies were diluted in block solution: pS6 ser 235/236 (Cell, Danvers, MA, USA D57.2.2E), 1:1000; S6 (Cell signaling, 5G10), 1:1000; pULK1 ser 757 (Cell signaling, D7O6U), 1:1000; ULK (Cell signaling, D8H5), 1:1000; LC3 (Cell signaling, #2775), 1:1000; β actin (Cell signaling, D6A8), 1:2000. The membranes, together with the primary antibodies, were incubated for 1 h at room temperature. After incubation, the membranes were washed five times with TBST. Then, antirabbit HRP at 1:3000 dilution (Cell signaling, #7074) was added. Five washes with TBST were performed to eliminate antibody excess. Images were obtained in ChemiDoc (BioRad, Hercules, CA, USA) and analyzed by Image Lab (BioRad, Hercules, CA, USA, version 6.1).

### 2.7. Statistical Analysis

Statistical analysis was performed through one- and two-way ANOVA and Bartlett’s post-analysis test, using GraphPad Prism software. The Student’s *t*-test was used for the bars, which represent standard deviations (SDs). Values of *p* < 0.05 were considered statistically significant. At least 2 independent experiments of n = 3 were conducted for each of the experimental processes.

## 3. Results

### 3.1. mTORC1 Activation by SopB Allows Salmonella Survival in B cells

**Figure 1:**
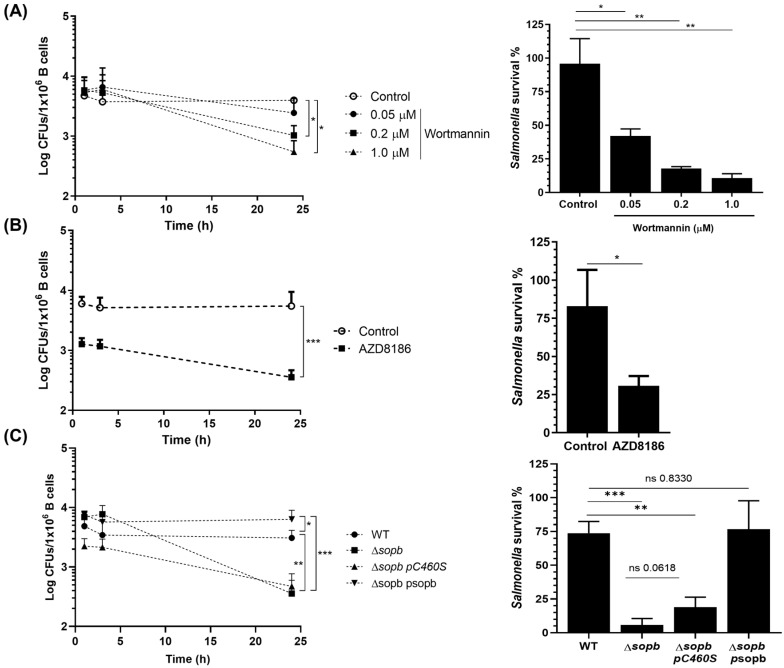
Class-I PI3K is needed for *Salmonella* survival in B cells. B cells were purified from the spleens of C57BL/6 mice and infected at a MOI of 50 with *Salmonella*. Subsequently, the cells were lysed and plated on LB Agar at the indicated times. (**A**) CFUs were recovered from infected B cells and treated 1 h before and during infection with the indicated concentrations of wortmannin or (**B**) treated with the PI3K class I inhibitor AZD8186 (500 nM). (**C**) B cells were infected at a MOI of 50 with *Salmonella* WT, *Salmonella* ∆sopb, *Salmonella* ∆sopb pC460S (phosphatase-inactive domain), or *Salmonella* ∆*sopb* psopb (WT SopB). Percentage of *Salmonella* survival in (**A**–**C**) was calculated by dividing CFUs recovered at 24 h between CFUs recovered at 1 h. Results are expressed as means ± SDs. n = 3. A two-way ANOVA test was used for multiple comparisons, and an unpaired Student’s *t*-test was used for bars. * *p* < 0.05, ** *p* < 0.01, and *** *p* < 0.001.

*Salmonella* activates the PI3K/Akt pathway during infection, and this activation allows *Salmonella* to inhibit pyroptosis in B cells, impeding the elimination of the bacteria. In these *Salmonella*-infected B cells, NLRP4 transcription and IL-1β production are inhibited. The pharmacological inhibition of PI3K or Akt, as well as the deletion of the rictor gene in B cells, is capable of rescuing the transcription of NLRP4 and IL-1β production, reflecting the control of *Salmonella* infection. These results suggest the possible activation of mTORC1 in *Salmonella*-infected B cells, blocking autophagy and avoiding the elimination of the bacteria [14,27,28]. Therefore, we considered whether, in addition to allowing the survival of B cells, PI3K/Akt activation participated in the inhibition of *Salmonella* infection control. For this purpose, we treated primary B cells with different concentrations of the PI3K class I inhibitor wortmannin during infection. The results showed that the control of *Salmonella* was proportional to PI3K inhibition by wortmannin (Figure 1A). We observed a similar result with AZD8186 (PI3K inhibitor), where B cells treated with this molecule substantially reduced their bacterial load (Figure 1B). None of the pharmacological inhibitors were toxic to B cells (Figure S2A) or *Salmonella* (Figure S2B) at the concentrations used. To corroborate the results obtained with the pharmacological inhibitors, we infected B cells with a *Salmonella* lacking the sopb gene (*Salmonella* Δ*sopb*) or with another strain complemented with a form of SopB lacking its inositide phosphatase function (*Salmonella* Δ*sopb* pC460S); both strains were incapable of activating the PI3K/Akt pathway. B cells infected with these mutant strains were able to control intracellular infections of *Salmonella* compared with B cells infected with *Salmonella* WT or with *Salmonella* Δ*sopb* complemented with sopb WT (*Salmonella* Δ*sopb* + psopb) (Figure 1C). Overall, these results showed that PI3K activation is essential for *Salmonella* survival in B cells.

**Figure 2:**
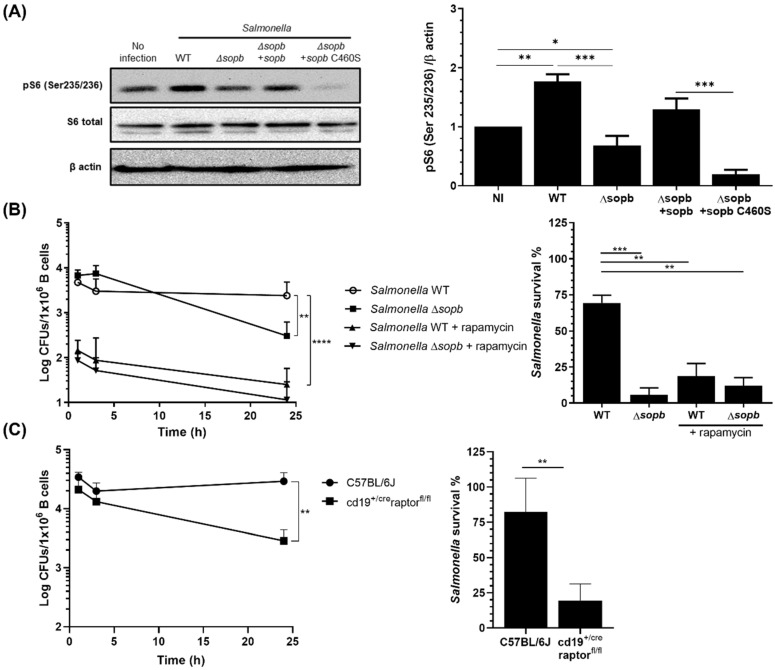
SopB activates mTORC1 to promote the survival of *Salmonella* in B cells. (**A**) mTORC1 activation was measured by pS6 (S235/S236) levels in B cells infected at a MOI of 50 with different strains of *Salmonella*. Total levels of 6S are shown. Data were normalized based on loading control and non-infected B cells. (**B**) CFUs recovered from B cells treated or not with the mTOC1 inhibitor rapamycin (160 nM) and infected at a MOI of 50 with *S*. Typhimurium WT or ∆sopb. (**C**) CFUs recovered from C57BL/6J or cd19^+/cre^raptor^fl/fl^ (mTORC1 KO) mouse B cells infected with *Salmonella* WT for 24 h. The percentage of *Salmonella* survival in (**B**) and (**C**) was calculated by dividing CFUs recovered at 24 h between CFUs recovered at 1 h. Results are expressed as means ± SDs. n = 3. A two-way ANOVA test was used for multiple comparisons and an unpaired Student’s *t*-test was used for bars. * *p* < 0.05, ** *p* < 0.01, *** *p* < 0.001 and **** *p* < 0.0001.

Since *Salmonella* induces the activation of the PI3K-Akt pathway in order to survive inside B cells, we evaluated whether *Salmonella* infection promotes the mTORC1 activation mediated by this pathway. For this purpose, we infected primary B cells for three hours with our different *Salmonella* strains; subsequently, we determined mTORC1 activation by measuring S6 protein phosphorylation. We observed an increase in pS6 in *Salmonella* WT-infected B cells compared with uninfected B cells. This increase did not occur in *Salmonella* Δ*sopb*-infected B cells (Figure 2A). By infecting B lymphocytes with *Salmonella* Δ*sopb* strains reconstituted with plasmids, as previously described, we determined that mTORC1 activation is mediated primarily by the inositide phosphatase function of SopB (Figure 2A). Once we determined that *Salmonella* activates mTORC1, we assessed whether this process is essential for *Salmonella* survival within B cells. For this purpose, we treated B lymphocytes with rapamycin (160 nM), an inhibitor of mTORC1. The rapamycin-treated B cells showed efficient control of *Salmonella* infection compared with the untreated B cells. *Salmonella* survival was affected in the presence of rapamycin in WT bacteria, as well as in the mutant (Δ*sopb*) (Figure 2B). This was reflected in the formation of CFUs and in the percentage of survival. In addition to the decrease we observed in the bacterial load at 24 h in rapamycin-treated B lymphocytes, we observed a substantial decrease in the numbers of *Salmonella* at 1 h post-infection (Figure 2B). The bacterial effector was therefore necessary for the maintenance of the bacteria in B cells. To corroborate the results obtained with rapamycin, we infected splenic B cells lacking mTORC1 (cd19^+/cre^raptor^fl/fl^) with *Salmonella.* The kinetics showed the effective control of *Salmonella* infection by B cells lacking mTORC1 compared with B cells from C57BL/6J mice (Figure 2C). These results are consistent with those obtained using rapamycin treatment. To reinforce the results obtained in vitro, as described above, cd19^+/cre^raptor^fl/fl^ mice were infected via orogastric inoculation with *Salmonella* WT GFP+. Preliminary results showed that cd19^+/cre^raptor^fl/fl^ mice had a lower bacterial load in the spleen and liver compared with WT mice. Additionally, when measuring the percentage of infection in mouse spleen B lymphocytes, a decrease in the percentage of CD19+ GFP+ cells was observed in cd19^+/cre^raptor^fl/fl^ mouse cells compared to what was seen in WT mouse cells (data not shown). Taking together, these results showed that *Salmonella*, through SopB, activates the mTORC1 complex, and that this activation allows *Salmonella* to survive intracellularly in B cells.

### 3.2. SopB-Mediated Activation of mTORC1 Inhibits Autophagy in B Cells

**Figure 3:**
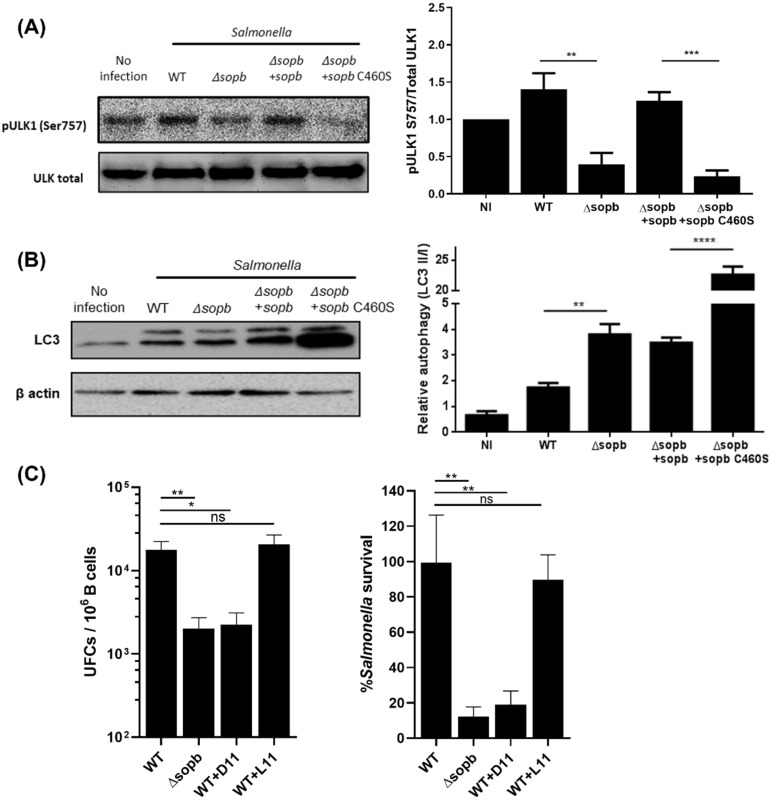
SopB-mediated activation of mTORC1 inhibits autophagy. Detection of pULK1 (S757) (**A**) or LC3-I/LC3-II (**B**) in B cells infected with indicated *Salmonella* strains for 3 h. (**C**) CFUs recovered from B cells infected with *Salmonella* strains for 24 h and treated for 1.5 h with autophagy-inducer peptide D11(20 μM) or scramble peptide L11 (20 μM). The percentage of *Salmonella* survival in (**C**) was calculated by considering *Salmonella* WT as 100% of survival. Results are expressed as means ± SDs. n = 3. An unpaired Student’s *t*-test was used for the bars. * *p* < 0.05, ** *p* < 0.01, *** *p* < 0.001, and **** *p* < 0.0001.

The PI3K/Akt/mTORC1 pathway is one of the central negative regulators of autophagy. Due to the fact that *Salmonella* activates and uses this pathway to survive in B cells, we evaluated whether *Salmonella* inhibits autophagy to promote its survival in B cells. For this purpose, we analyzed the phosphorylation of ULK1 Ser757; this phosphorylation is provided by mTORC1 and prevents autophagy initiation. *Salmonella* WT infection increased the levels of pULK1 Ser757 in B lymphocytes compared with uninfected cells. In contrast, *Salmonella* Δ*sopb* infection did not induce this increase in pULK1 Ser757 (Figure 3A). Using reconstitution assays with psopb and psopb C460S plasmids, we determined that the inositide phosphatase function is responsible for the increase in pULK1 Ser757. We measured the LC3-II/LC3-I levels to corroborate the decrease in autophagy during *Salmonella* infection. The LC3-II/LC3-I levels showed a pattern similar to that of pULK1 Ser757; the autophagy levels decreased in B cells infected with *Salmonella* WT in contrast with the levels we observed in B cells infected with *Salmonella* Δ*sopb*. Likewise, SopB, through its inositide phosphatase function, was mainly responsible for the observed effect (Figure 3B). The obtained results indicated that *Salmonella* inhibits autophagy in B cells. To determine whether this mechanism is essential for *Salmonella* survival, we treated infected B cells with D11, an autophagy-inducer peptide. Whereas untreated B cells were unable to control *Salmonella* infection, B cells treated with D11 efficiently controlled *Salmonella* WT, in a manner similar to that which we observed in B cells infected with Salmonella Δ*sopb*. As expected, we observed a recuperation of the percentage of surviving bacteria when treated with L11, a scramble peptide for D11, up to the percentage observed in the survival of *Salmonella* WT (Figure 3C). This result demonstrated that B cells, through autophagy, control *Salmonella* infection; however, the bacteria are capable of surviving within B cells that inhibit autophagy.

## 4. Discussion

The interplay between *Salmonella* and autophagy has been addressed mainly in HeLa cells and macrophages [28,29,30,31,32]. However, the mechanism *Salmonella* uses during infection of target B cells and the mechanisms that allow the survival of this bacterium in B cells have not been addressed in depth.

Here, we have shown, for the first time, that the *Salmonella* effector protein SopB activates mTORC1 and inhibits autophagy by phosphorylating ULK1 at its Ser757. The activation of this pathway facilitates *Salmonella* survival in B cells. The inhibition of autophagy in *Salmonella*-infected B cells contrasts with the findings reported for epithelial cells, where *Salmonella* proliferation is controlled by autophagy [33]. In HeLa cells, *Salmonella* is labeled by NDP52 and recruited to autophagosomes by galectin 8 (GAL8). Similarly, in macrophages, compartments containing *Salmonella* are labeled by LC3. In both cases, these labels favor bacteria control by autophagy [34,35]. The accumulation of LC3-II levels, as shown in Figure 3A, resembles what is observed in cells treated with chloroquine (CQ) and bafilomycin A (BafA). These molecules impair the function of lysosomes [33,34]. SopB might also block lysosome function; nevertheless, *Salmonella*-infected B cells are capable of completing the autophagy process when treated with autophagy inductor D11, thus controlling infection. The pULK1 levels were not statistically different in *Salmonella* WT-infected B cells compared with those observed in *Salmonella*-complemented strain-infected lymphocytes (Figure 3B). These results contrast with our observation that levels of the LC3-II/LC3-I ratio differ between the two groups of cells infected either with the *Salmonella* Δ*SopB* complemented strain or *Salmonella* WT. Other bacterial effectors likely participate in infected B cells along the autophagy pathway.

The activation of the PI3K/Akt/mTORC1 pathway by *Salmonella* was previously reported in macrophages. However, the bacterial effector responsible for such effects was not reported [29]. The hypothesis that SopB in B cells is responsible for the activation of the PI3K/Akt/mTORC1 pathway is supported both by previous reports [13,14] and by the actual results obtained in SopB-complemented assays, in which the SopB inositide phosphatase function was found to be responsible for the activation of this signaling pathway (Figure 2 and Figure 3). This can occur in infected B cells as well as in neighboring B cells, as previously described for the downregulation of NLRC4 levels [13] and the accumulation of PIP3 in the cytoplasmic membrane [14], which is observed in infected and noninfected B cells.

B cells substantially increased autophagy levels in response to *Salmonella* Δ*sopb* or *Salmonella* Δ*sopb* pC460S infection (Figure 3). These results were probably due to AMPK activation being promoted by the decrease in cytoplasmic amino acid concentration, in a manner similar to that which was observed in *Salmonella*-infected HeLa cells [35]. In *Salmonella*-infected B cells, autophagy was blocked due to the SopB-mediated activation of mTORC1, causing the infected cells to maintain levels similar to those of uninfected B cells (Figure 3). Since autophagy is an essential process for maintaining cell homeostasis, autophagy levels during infection that remain close to those observed for basal levels may favor the functional maintenance of B cells and prevent their death.

Although the data suggested that, in B cells, SopB is mainly responsible for blocking autophagy, in other cell lines, other bacterial effectors triggered this phenomenon, as is the case for the macrophage cell line J774A.1, in which the phosphothreonine lyase spvC is responsible for inhibiting autophagy [28]. This result suggested that *Salmonella* possess several mechanisms that allow modification to mammalian cell biology and adapt according to host cell lineage.

As one of the consequences of mTORC1 activation, we observed phosphorylation of ULK1 at Ser757 and thus blocking of autophagy (Figure 2 and Figure 3). However, due to the high number of signaling pathways impacted by mTORC1, we considered that SopB may be modifying other signaling pathways, as well as the metabolic state of B cells, thereby favoring the establishment of appropriate modifications in B cells, permitting them to become promising niches for *Salmonella*.

**Figure 4:**
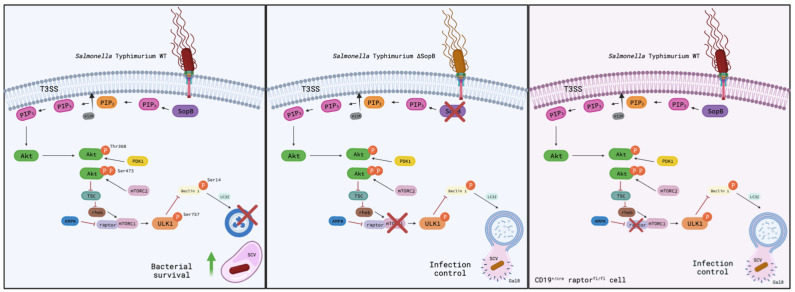
*Salmonella* promotes its survival in B cells through SopB-mediated autophagy inhibition. During B cell infection, *Salmonella* translocate SopB, which mediates, through its inositide phosphatase domain, the induction of the activation of PI3K/Akt/mTORC1 pathway. As a result of mTORC1 activation, ULK1 is phosphorylated at its ser 757, inhibiting the autophagy process and allowing the survival of *Salmonella* in these cells (left). In the absence of SopB, *Salmonella* is unable to activate the PI3K/Akt/mTORC1 pathway, permitting increased levels of autophagy (including the fusion of the autophagosome with the Salmonella-containing vacuole “SCV” marked with galectin-8 for its recognition) and the control of the bacterium (center). This last phenomenon can be seen in cd19^+/cre^ raptor^fl/fl^ (mTORC1 KO) mice, where the absence of the protein raptor disables the formation of the mTORC1 complex, thus inhibiting the autophagy process.

Our results suggested that B lymphocytes may be important niches in which *Salmonella* remains viable long enough to achieve a persistent infection and invade other types of tissues. Evaluating the function of *Salmonella-*infected B cells with the objective of better understanding the likely effect on antibody production would be interesting. Overall, our results demonstrated that *Salmonella* survival within B cells depends on the inhibition of autophagy by mTORC1 activation (Figure 4).

## Supplementary Materials

The following supporting information can be downloaded at: https://www.mdpi.com/article/10.3390/cells11132061/s1, Figure S1: Purified B lymphocytes by MACs and Salmonella infected purified B cells; Figure S2: Pharmacological inhibitors do not affect the viability of B cells or Salmonella; Figure S3: Characterization of cd19+/creraptorfl/fl mice.

## References

[1] Haraga A., Ohlson M.B., Miller S.I. (2008). Salmonellae interplay with host cells. Nat. Rev. Microbiol..

[2] Centers for Disease Control and Prevention (2019). Drug Resistant Nontyphoidal Salmonella. www.wou.edu/las/natsci_math/biology/boomer/Bio440/emerging2002/Salmonella2.

[3] Levine M.M., Black R., Lanata C. (1982). Precise Estimation of the Numbers of Chronic Carriers of Salmonella typhi in Santiago, Chile, an Endemic Area. J. Infect. Dis..

[4] Hornick R.B., Greisman S.E., Woodward T.E., DuPont H.L., Dawkins A.T., Snyder M.J. (1970). Typhoid Fever: Pathogenesis and Immunologic Control. N. Engl. J. Med..

[5] Dongol S., Thompson C.N., Clare S., Nga T.V.T., Duy P.T., Karkey A., Arjyal A., Koirala S., Khatri N.S., Maskey P. (2012). The Microbiological and Clinical Characteristics of Invasive Salmonella in Gallbladders from Cholecystectomy Patients in Kathmandu, Nepal. PLoS ONE.

[6] Brigo N., Pfeifhofer-Obermair C., Tymoszuk P., Demetz E., Engl S., Barros-Pinkelnig M., Dichtl S., Fischer C., Valente De Souza L., Petzer V. (2021). Cytokine-Mediated Regulation of ARG1 in Macrophages and Its Impact on the Control of *Salmonella enterica* Serovar Typhimurium Infection. Cells.

[7] Rosales-Reyes R., Alpuche-Aranda C., Ramírez-Aguilar M.D.L.L., Castro-Eguiluz A.D., Ortiz-Navarrete V. (2005). Survival of *Salmonella enterica* Serovar Typhimurium within Late Endosomal-Lysosomal Compartments of B Lymphocytes Is Associated with the Inability To Use the Vacuolar Alternative Major Histocompatibility Complex Class I Antigen-Processing Pathway. Infect. Immun..

[8] Castro-Eguiluz D., Pelayo R., Rosales-Garcia V., Rosales-Reyes R., Alpuche-Aranda C., Ortiz-Navarrete V. (2009). B cell precursors are targets for Salmonella infection. Microb. Pathog..

[9] Alvarez M.I., Glover L.C., Luo P., Wang L., Theusch E., Oehlers S.H., Walton E.M., Tram T.T.B., Kuang Y.-L., Rotter J.I. (2017). Human genetic variation in *VAC14* regulates *Salmonella* invasion and typhoid fever through modulation of cholesterol. Proc. Natl. Acad. Sci. USA.

[10] Patel J.C., Galán J.E. (2006). Differential activation and function of Rho GTPases during Salmonella–host cell interactions. J. Cell Biol..

[11] Burkinshaw B.J., Prehna G., Worrall L.J., Strynadka N.C. (2012). Structure of Salmonella Effector Protein SopB N-terminal Domain in Complex with Host Rho GTPase Cdc42. J. Biol. Chem..

[12] Knodler L., Finlay B.B., Steele-Mortimer O. (2005). The Salmonella Effector Protein SopB Protects Epithelial Cells from Apoptosis by Sustained Activation of Akt. J. Biol. Chem..

[13] Perez-Lopez A., Rosales-Reyes R., Alpuche-Aranda C.M., Ortiz-Navarrete V. (2013). *Salmonella* Downregulates Nod-like Receptor Family CARD Domain Containing Protein 4 Expression To Promote Its Survival in B Cells by Preventing Inflammasome Activation and Cell Death. J. Immunol..

[14] García-Gil A., Galán-Enríquez C.S., Pérez-López A., Nava P., Alpuche-Aranda C., Ortiz-Navarrete V. (2018). SopB activates the Akt-YAP pathway to promote *Salmonella* survival within B cells. Virulence.

[15] Castrejón-Jiménez N.S., Leyva-Paredes K., Hernández-González J.C., Luna-Herrera J., García-Pérez B.E. (2015). The role of autophagy in bacterial infections. Biosci. Trends.

[16] Ogawa M., Yoshimori T., Suzuki T., Sagara H., Mizushima N., Sasakawa C. (2005). Escape of Intracellular *Shigella* from Autophagy. Science.

[17] Kim J., Kundu M., Viollet B., Guan K.-L. (2011). AMPK and mTOR regulate autophagy through direct phosphorylation of Ulk1. Nat. Cell Biol..

[18] Starokadomskyy P., Dmytruk K.V. (2013). A bird’s-eye view of autophagy. Autophagy.

[19] Meley D., Bauvy C., Houben-Weerts J.H., Dubbelhuis P.F., Helmond M.T., Codogno P., Meijer A.J. (2006). AMP-activated Protein Kinase and the Regulation of Autophagic Proteolysis. J. Biol. Chem..

[20] Zhu Y.P., Brown J.R., Sag D., Zhang L., Suttles J. (2014). Adenosine 5′-Monophosphate–Activated Protein Kinase Regulates IL-10–Mediated Anti-Inflammatory Signaling Pathways in Macrophages. J. Immunol..

[21] Birmingham C.L., Canadien V., Kaniuk N.A., Steinberg B.E., Higgins D.E., Brumell J.H. (2008). Listeriolysin O allows Listeria monocytogenes replication in macrophage vacuoles. Nature.

[22] Huang D., Bao L. (2015). Mycobacterium tuberculosis EspB protein suppresses interferon-γ-induced autophagy in murine macrophages. J. Microbiol. Immunol. Infect..

[23] Kayath C.A., Hussey S., El Hajjami N., Nagra K., Philpott D., Allaoui A. (2010). Escape of intracellular Shigella from autophagy requires binding to cholesterol through the type III effector, IcsB. Microbes Infect..

[24] Miao E., Brittnacher M., Haraga A., Jeng R.L., Welch M.D., Miller S.I. (2003). Salmonella effectors translocated across the vacuolar membrane interact with the actin cytoskeleton. Mol. Microbiol..

[25] Knodler L.A., Winfree S., Drecktrah D., Ireland R., Steele-Mortimer O. (2009). Ubiquitination of the bacterial inositol phosphatase, SopB, regulates its biological activity at the plasma membrane. Cell. Microbiol..

[26] Finn C.E., Chong A., Cooper K.G., Starr T., Steele-Mortimer O. (2017). A second wave of Salmonella T3SS1 activity prolongs the lifespan of infected epithelial cells. PLOS Pathog..

[27] Rosales-Reyes R., Pérez-López A., Sánchez-Gómez C., Hernández-Mote R.R., Castro-Eguiluz D., Ortiz-Navarrete V., Alpuche-Aranda C.M. (2012). Salmonella infects B cells by macropinocytosis and formation of spacious phagosomes but does not induce pyroptosis in favor of its survival. Microb. Pathog..

[28] Zhou L., Li Y., Gao S., Yuan H., Zuo L., Wu C., Huang R., Wu S. (2021). Salmonella spvC Gene Inhibits Autophagy of Host Cells and Suppresses NLRP3 as Well as NLRC4. Front. Immunol..

[29] Owen K.A., Meyer C.B., Bouton A.H., Casanova J.E. (2014). Activation of Focal Adhesion Kinase by Salmonella Suppresses Autophagy via an Akt/mTOR Signaling Pathway and Promotes Bacterial Survival in Macrophages. PLOS Pathog..

[30] Scheidel J., Amstein L., Ackermann J., Dikic I., Koch I. (2016). In Silico Knockout Studies of Xenophagic Capturing of Salmonella. PLOS Comput. Biol..

[31] Kreibich S., Emmenlauer M., Fredlund J., Rämö P., Münz C., Dehio C., Enninga J., Hardt W.-D. (2015). Autophagy Proteins Promote Repair of Endosomal Membranes Damaged by the Salmonella Type Three Secretion System 1. Cell Host Microbe.

[32] Cemma M., Kim P.K., Brumell J.H. (2011). The ubiquitin-binding adaptor proteins p62/SQSTM1 and NDP52 are recruited independently to bacteria-associated microdomains to target Salmonella to the autophagy pathway. Autophagy.

[33] Mauthe M., Orhon I., Rocchi C., Zhou X., Luhr M., Hijlkema K.-J., Coppes R.P., Engedal N., Mari M., Reggiori F. (2018). Chloroquine inhibits autophagic flux by decreasing autophagosome-lysosome fusion. Autophagy.

[34] Fedele A.O., Proud C.G. (2020). Chloroquine and bafilomycin A mimic lysosomal storage disorders and impair mTORC1 signalling. Biosci. Rep..

[35] Tattoli I., Sorbara M.T., Vuckovic D., Ling A., Soares F., Carneiro L., Yang C., Emili A., Philpott D.J., Girardin S.E. (2012). Amino Acid Starvation Induced by Invasive Bacterial Pathogens Triggers an Innate Host Defense Program. Cell Host Microbe.

