# Supplementary material for: Salmonella Promotes Its Own Survival in B Cells by Inhibiting Autophagy
Source: Cells. 2022 Jun 29;11(13):2061. doi: 10.3390/cells11132061 (PMC9266210; doi:10.3390/cells11132061)
Supplement: Supplementary file 1 [file cells-11-02061-s001.zip › cells-1714119-supplementary.pdf]

Supplementary material

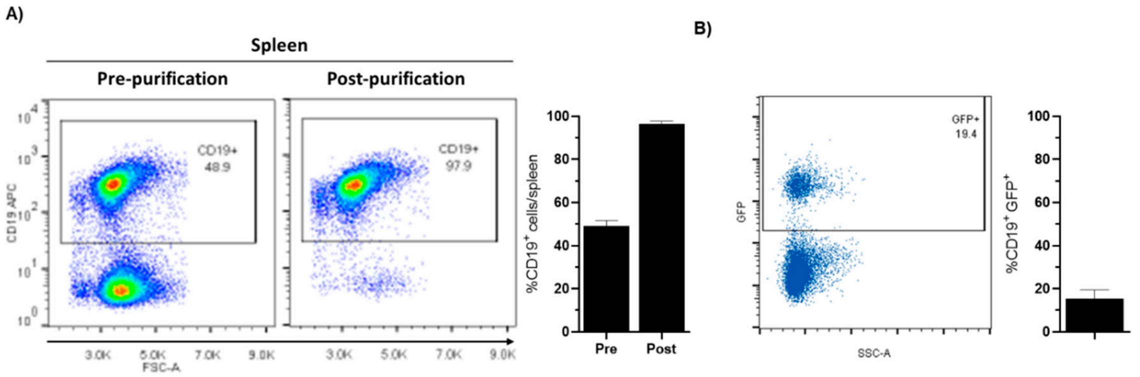

Figure S1. Purified B lymphocytes by MACs and *Salmonella* infected purified B cells. (A) Representative plots of mice splenocytes before and after purification done by negative selection. A previous singlets gating was performed for data analysis. (B) The percentage of B cells infected with *Salmonella* was evaluated by flow cytometry using a GFP^+ strain of *Salmonella*. A representative plot is shown. Population analysis was done starting with the region of singlets, followed by the selection of the mononuclear region and finally the $CD19^+$ region, corresponding to B cells population. Results are expressed as mean ± SD, n=5.

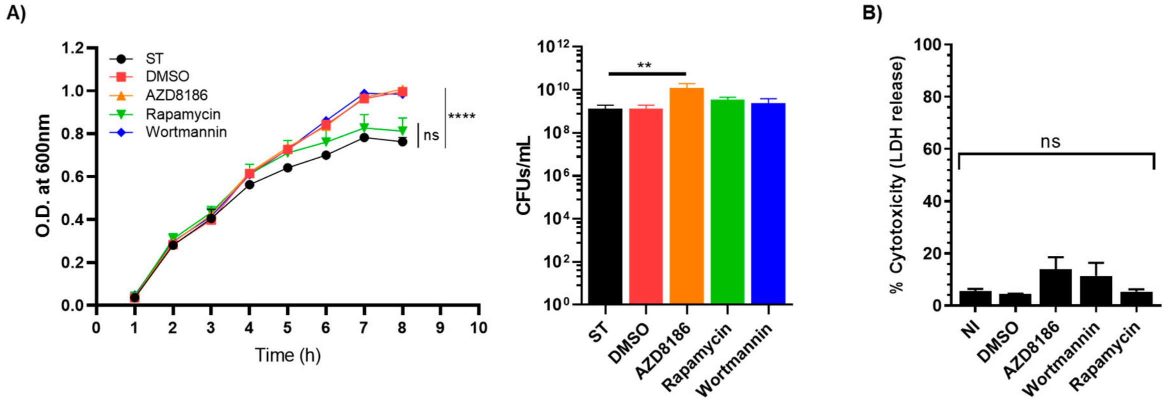

Figure S2. Pharmacological inhibitors do not affect the viability of B cells or *Salmonella*. (A) *Salmonella* was cultured in LB medium added with DMSO (42 mM), AZD8186 (500 nM), rapamycin (160 nM) or wortmannin (1.0 μM). Bacterial growth was evaluated each hour for 8 hours by optical density at 600 nm. Once the incubation time was done, CFUs were calculated by plating dilutions of 10^{-6}, 10^{-7} and 10^{-8} on LB agar. (B) B cells were incubated with the pharmacological inhibitors at the same concentration as in (A) and their cytotoxicity was evaluated through LDH release. Results are expressed as mean ± SD, n=4 for (A) and n=3 for (B). A one-way ANOVA test was used for multiple comparisons and corrected through Bartlett's test. * P<0.05, ** P<0.01 and *** P<0.001.

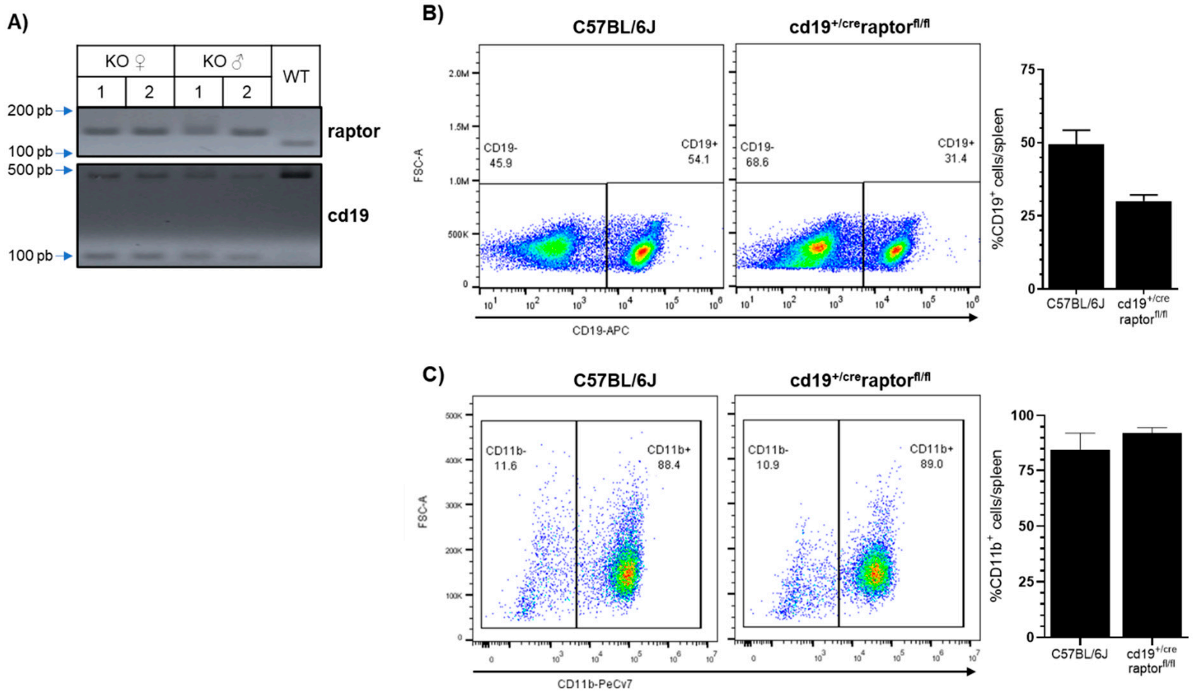

Figure S3. Characterization of cd19$^{+/cre}$raptor$^{fl/fl}$ mice. (A) Mice genotype was corroborated by PCR according to Jackson Laboratory protocol. PCR products were resolved on 3% agarose gel. A band of 140 bp indicates the presence of the *raptor* WT gene while a band of 180 bp is indicative of raptor^{fl} gene. For the *cd19* WT gene a band of 477 bp is indicative of its presence and a band of 100 bp is visible in the case of cd19^{cre} mice. (B) The percentage of C56BL/6J and cd19$^{+/cre}$ raptor$^{fl/fl}$ mice B cells was evaluated by flow cytometry. A representative plot is shown. (C) Analogous to this the percentage of macrophages in C56BL/6J and cd19$^{+/cre}$ raptor$^{fl/fl}$ mice was evaluated as described in (B). Results are expressed as mean ± SD, n=5.
